# Keratoconus: Tissue Engineering and Biomaterials

**DOI:** 10.3390/jfb5030111

**Published:** 2014-09-11

**Authors:** Dimitrios Karamichos, Jesper Hjortdal

**Affiliations:** 1 Department of Ophthalmology, University of Oklahoma Health Sciences Center, 608 Stanton L. Young Blvd, DMEI PA-409, Oklahoma City, OK 73104, USA; 2 Department of Ophthalmology, Aarhus University Hospital, Aarhus C DK-800, Denmark; E-Mail: jesper.hjortdal@dadlnet.dk

**Keywords:** keratoconus, *in vitro*, corneal cells, tissue engineering, biomaterials

## Abstract

Keratoconus (KC) is a bilateral, asymmetric, corneal disorder that is characterized by progressive thinning, steepening, and potential scarring. The prevalence of KC is stated to be 1 in 2000 persons worldwide; however, numbers vary depending on size of the study and regions. KC appears more often in South Asian, Eastern Mediterranean, and North African populations. The cause remains unknown, although a variety of factors have been considered. Genetics, cellular, and mechanical changes have all been reported; however, most of these studies have proven inconclusive. Clearly, the major problem here, like with any other ocular disease, is quality of life and the threat of vision loss. While most KC cases progress until the third or fourth decade, it varies between individuals. Patients may experience periods of several months with significant changes followed by months or years of no change, followed by another period of rapid changes. Despite the major advancements, it is still uncertain how to treat KC at early stages and prevent vision impairment. There are currently limited tissue engineering techniques and/or “smart” biomaterials that can help arrest the progression of KC. This review will focus on current treatments and how biomaterials may hold promise for the future.

## 1. Introduction

Keratoconus (KC) is a bilateral degenerative non-inflammatory corneal disorder with prevalence of 1 in 2000 people worldwide, although this number varies considerably between studies [1,2,3,4,5,6,7,8,9,10]. While KC is observed in populations throughout the world, it is reported more frequently in certain ethnic groups such as South Asians, Eastern Mediterranean, and North Africans [11,12,13]. The incidence is believed to be as high as 1 in 500 [2] but difficulties with differential diagnosis causes uncertainty as to its prevalence. KC is known to typically initiate at puberty and progress until the third or fourth decade when it usually arrests [14,15,16]. The rate of the progression varies significantly between individuals and not everyone will experience severe stages of the disease. It is estimated that 10%–15% of KC diagnosed patients will reach severe stages and require corneal transplantation in order to have functional vision [1,2,3,4,5,6,7,8,9,10,11,12,13,14,15,16]. 

At early stages, patients typically experience minor blurring with the symptoms being identical to refractive defect and irregular astigmatism [3,17,18,19]. As KC progresses, vision deteriorates. The degree of vision impairment depends on the rate of progression. At mid-stages, KC is easily diagnosed and patients experience trouble with their night vision, photophobia, eye strain, and eye itching [20,21,22,23]. Most of the time, advanced KC stages develop corneal scarring which contributes to further vision loss and ultimately makes corneal transplantation necessary. 

This disease has a profound effect on patients and may result in significant difficulties with conducting every day activities. Previous reviews have concentrated on management of the disease and clinical/surgical options [3,24,25,26,27,28,29,30] and will be discussed briefly here. The reality is that most of the treatments are available to improve the quality of life but not necessarily to treat the disease. Even with the recent success of collagen cross-linking with riboflavin (or CXL), there are still questions with regard to its long-term efficiency. CXL was first introduced in Europe about 10 years ago and is currently in clinical trials in USA [31,32,33,34,35]. Time will only tell whether this technology arrests the disease for life or just delays the process. 

The purpose of this review is to outline current techniques to arrest KC disease and discuss promising tissue engineering techniques and biomaterials that are available but have never been tested on KC. 

## 2. Pathophysiology and Etiology

It is widely believed that genetics, the environment, and the cellular mechanism all play a role in KC [9,36,37,38,39,40,41,42,43,44,45,46,47,48,49]. However, the exact contribution of each of the above to the etiology of KC is unknown. It is almost certain that KC is a multifactorial disease and the onset is still a mystery. KC has its onset at puberty and it can progress until the third or fourth decade of life; however, it can arrest at any point [3,50]. While multiple reports have associated KC with other disorders, it is more commonly seen as an isolated condition. The most common disorders associated with KC are Down syndrome and Leber’s congenital amaurosis [40,51,52,53,54]. Rabinowitz’s 1998 review discussed these studies [3]. In some cases, KC appears to have a familial association. However, in a study at the Cedars-Sinai Medicine Center, authors found that 99% of the 300 KC patients had no association with genetic diseases [3,54,55].

In terms of sex preference of the disease, it seems to affect both male and female [56]. In fact, female or male dominance is unclear based on data reported from various studies. Some studies report a preponderance of men over women and others report the exact opposite [5,7,56,57,58,59,60,61,62,63]. It is known, however, that higher numbers of KC disorders are seen in the South Asian region. 

One of the most common associations of KC is eye rubbing [59,64,65,66,67,68]. This environmental cause was first introduced by Ridley who discovered the relationship between KC and atopic disease [69]. In Ridley’s study, more that 70% of KC patients vigorously rubbed their eyes [69]. Further support of this theory was provided by subsequent studies [70]. Other environmental factors include poorly fit contact lenses and allergies [70,71,72,73]. The relationship between KC and contact lenses or allergies is still questioned by scientists and further studies are needed. In both cases, however, eye rubbing is a possible confounder.

Cellular dysfunction and biochemical abnormalities are almost certain to play a role in KC onset as well as progression. Various authors have suggested abnormalities in collagen fibers within the cornea and their cross-linking. Others have reported abnormalities in proteoglycans and proteoglycan metabolism of the cornea [41,42,43,44,45,46,47,48,49]. One recent discovery is the abnormal processing of superoxide radicals in KC corneas and the involvement of oxidative stress in KC [74,75,76,77]. This is now linked to the quality of tears and the disruption on collagen structure due to the creation of harmful byproducts of cell metabolism.

Overall, there are many potential candidates for the onset and progression of the KC disease. These candidates (genetic, environmental, cellular, or other factors) may be acting alone or in combination, leading to a vision threatening condition.

## 3. Clinical Characteristics and Management

The disease may progress very fast or very slow or may even stop at early stages without any further complications. In any scenario, patients that reach severe stages require some kind of clinical intervention. Below, we briefly discuss the three main options available to patients. Other options are available and have been reviewed elsewhere [1,40,78,79,80,81,82,83,84,85,86,87,88,89,90,91,92,93,94,95,96,97,98,99,100,101,102,103]. 

### 3.1. Penetrating Keratoplasty (PKP)

**Figure 1:**
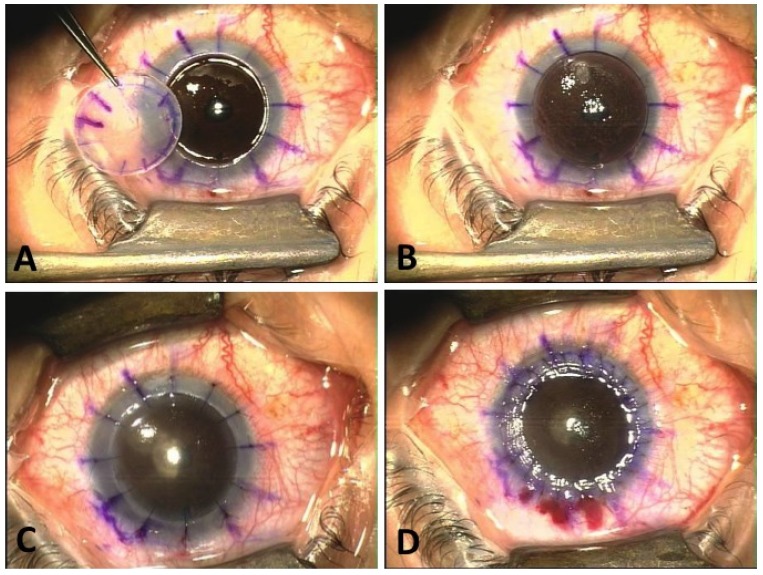
Penetrating keratoplasty. (**A**) Distorted cornea removed; (**B**) Corneal graft placed in recipient bed; (**C**) “Stay sutures” placed; (**D**) Single running suture in place at end of surgery.

It is estimated that approximately 10%–15% of KC cases will progress to an extent which requires surgical intervention [1,100,101,104]. Once the cornea becomes excessively ectatic, thin or scarred, no correction can help the patient. Contact lenses cannot be worn or will not improve visual acuity and therefore corneal transplantation is required. Overall, penetrating keratoplasty (or PKP) is the most common procedure (Figure 1) for individuals with severe KC [105]. Generally, PKP is a successful procedure for KC patients, with favorable results [1,106,107,108,109]. Niziol (2013) [108] recently reported a 90% success rate for 5–12 year-old grafts. There have been multiple studies with various populations and number of patients, such as from Lim and co-authors [110], reporting a success rate of 95.7% from a total of 93 eyes receiving PKP [110,111]. From the recent literature, it seems that graft survival is improving. In 1972, Keates *et al*. [111] reported a success rate slightly over 80%. A little later, Troutman and co-authors reported an 88.4% success rate [112]. The acute recovery of someone receiving PKP is anywhere between 4 and 6 weeks, but stable vision is not achievable for at least a year post-op [101,105]. Any complications following PKP are related to vascularization and rejection of the donor cornea. Other known but rare complications, are cataract, loose suture, glaucoma, and severe astigmatism [113,114,115,116,117]. Overall, this is a safe surgical option which is heavily used by surgeons. The huge drawback is the availability of donor corneas, especially in under-developed countries.

### 3.2. Deep Anterior Lamellar Keratoplasty (DALK)

**Figure 2:**
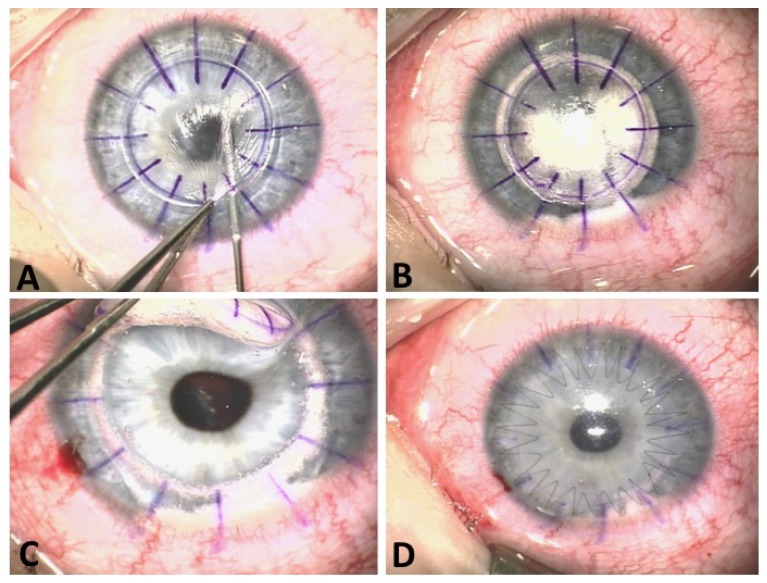
Deep anterior keratoplasty (DALK). (**A**) After partial trephination, a cannula is inserted deep in the corneal stroma; (**B**) Air injection through the cannula separating Descemets membrane from stroma; (**C**) Manual dissection ensuring that only the recipient’s Descemets membrane is preserved; (**D**) Lamellar donor graft sutured in place at end of surgery.

Deep anterior lamellar keratoplasty (DALK) is a relatively new technique and so far it has been very promising [118]. In DALK (Figure 2), surgeons remove the corneal epithelium and stroma, but not the endothelium, from the host cornea. This is a major advantage over PKP since preservation of host endothelium reduces the risk of graft rejection. The actual surgery is challenging and requires expertise. In fact, a variety of DALK modifications have been reported and recently reported by Fogla [119], including “peeling technique” [120], intrastromal air injection [121], and hydro-delamination [122]. Our data on DALK is rather premature since it was only introduced to keratoconus patients in the mid-90s [123,124,125,126]. However, the post-op results so far have been promising. An average of 80% of KC patients showed 20/40 visual acuity after receiving DALK [127,128,129]. For many surgeons, DALK is the “next big thing” and an alternative to PKP. DALK significantly minimizes common PKP complications such as wound leakage and endothelial graft rejection, but Descement membrane perforation [122,129,130,131,132], secondary anterior chamber formation [133], and interface keratitis [134] can develop as a result of DALK.

### 3.3. Intrastromal Ring Segments (INTACS)

**Figure 3:**
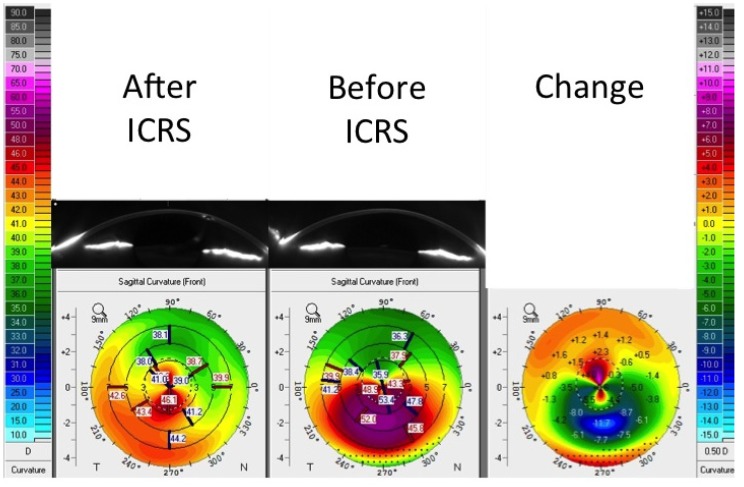
Intracorneal ring segments. Scheimpflug image inserts show keratoconic cornea before and after insert of one intracorneal ring segment. Color images show corneal power before and after a segment insert and the change in corneal power. Note that corneal power after segment implantation is more regular.

Intrastromal ring segments (INTACS) are polymethyl methacrylate and acrylic polymer inserts that were originally designed for myopia correction purposes [135,136,137,138,139,140]. Currently, they are used as an effective treatment for mild to moderate keratoconus [135,141,142,143,144,145,146,147,148,149,150,151,152,153,154,155,156,157,158,159]. The major function of INTACS is to reshape KC corneas to a more regular cornea surface and allow better contact lens fitting and therefore improve vision [141,160]. During an INTACS operation, no cornea tissue is removed and overall, the surgery is less invasive. INTACS are placed within the cornea (one on each side) to lift the inferior or superior ectasia and flatten the bulged cornea area of KC [141,160]. Colin and co-authors [161] were the first to apply INTACS to KC patients. Since then, there have been a number of studies reporting results on INTACS [135,143,144,146,147,148,149,150,151,152,153,154,155,156,157,158,159,162]. Overall, INTACS are a good option in order to temporarily improve contact lens fitting and vision (Figure 3). INTACS are not used as a treatment to arrest KC disease, but they hold a lot of promise in delaying the need for PKP and other invasive surgical solutions.

### 3.4. Corneal Crosslinking (CXL)

**Figure 4:**
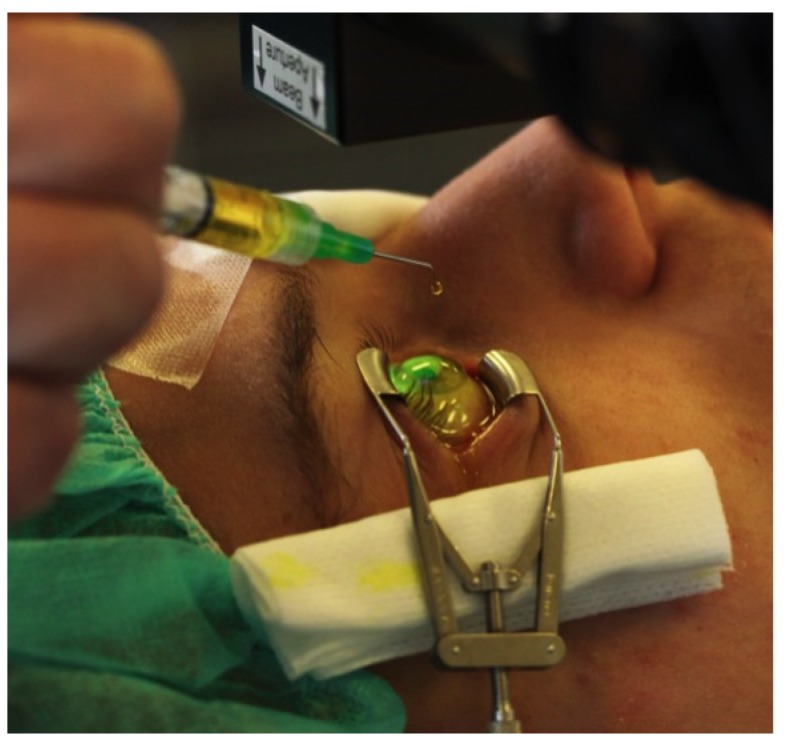
Application of riboflavin drops during corneal cross linking procedure.

The potential of ultraviolet-A light (UVA) to crosslink tissues in the presence of riboflavin has been known for some time. However, it was not until 1998 that this strategy was proposed as a therapeutic corneal treatment. Since then, interventions have been performed in Europe, while in the USA clinical trials were initiated in 2008 and are awaiting FDA approval. The procedure is relatively easy and well documented. Briefly, CXL is achieved via application of riboflavin solution (Figure 4) over 30 min on the de-epithelialization cornea, followed by UV-A illumination for approximately 30 min or less [163,164,165,166,167]. Activated riboflavin results in the formation of new bonds across adjacent corneal stroma collagen strands and ground substance, ultimately leading to strengthening of corneal stroma mechanics. In case of KC, this is currently used routinely in Europe and the treatment in most cases arrests the disease progression [168,169,170,171,172,173,174]. The routinely used technique is performed with the epithelium layer removed from the corneal stroma in order to ensure better riboflavin penetration. Currently, there are a number of studies looking into riboflavin penetration with epithelium intact and how this may improve CXL [163,165,167,174,175,176,177,178]. These have been previously reviewed and will not be discussed here [163]. Overall, CXL is a promising technique to arrest the disease, but we still lack long-term data [167,168,169,170,171,172,173,174]. 

## 4. *In Vitro* Strategies

The World Health Organization (WHO) reported that corneal diseases are a major cause of vision loss and blindness, second only to cataracts in overall importance [10]. The only acceptable method for treating corneal blindness is by transplantation with matched donor tissue. It is easily understood that the demand for donor corneas exceeds the supply and therefore other strategies have been studied. One of them is *in vitro* models. Can corneas be grown in the lab from human cells and then be transplanted to a patient with trauma or blindness? Despite recent advancements in designing those tissues *in vitro*, there are very few that have come close. This is not a surprise, considering the complex nature of the corneal tissue. An *in vitro* tissue would have to have at least the stromal layer in place, if not the stroma and epithelium, the right thickness, with the correct organization, immune properties, and optical characteristics. Of the models developed so far, probably the most promising was the model by the Laboratoire d’Organogenese Experimentale (LOEX) [3,6]. The authors used the self-assembly approach where cells were stimulated with ascorbic acid to induce ECM production. They were able to stack multiples of those 3D ECMs and seed an epithelium on top and an endothelium at the bottom. This was well received with good tensile strength, but no optical data was reported [3,6]. In KC, an approach like this would not be necessary since the endothelium normally remains intact and what needs to be restored are the stroma and/or epithelium. In a recent study, corneal stromal cells derived from KC donors were isolated and stimulated with ascorbic acid [4] in order to characterize ECM secretion and assembly. The authors reported major differences from healthy corneal cells including the inability to secrete large amounts of ECM, higher number of myofibroblasts, and fibrotic ECM [4]. This confirmed the dysfunction of KC stromal cells and the need for replacement or reprogramming. It is therefore clear that the 3D *in vitro* models hold great potential for KC transplantation, but more refinement and studies are necessary before those can be transplanted. Other *in vitro* models of KC disease are concentrating on 2D studies and cell–cell interactions [2,5,6,7,8,9,11,12,13,14,15,16]. While these are invaluable and provide a better understanding on mechanism, the more clinically oriented models are the ones that can possibly provide a future treatment of the KC defects. 

## 5. Tissue Engineering Materials

Tissue engineering strategies have been very important in a variety of therapeutic approaches [179,180,181,182,183,184]. The ultimate goal is to repair or replace portions or whole tissues using these strategies. KC is a corneal dystrophy that currently lacks animal model for detailed studies and interestingly enough not many tissue engineering strategies have been attempted. It is important to keep in mind a few rules about using tissue engineering solutions for corneal tissue. An artificial cornea would have to adhere to several basic rules in order to be successful. (1) Integrate into the recipient tissue and the surroundings, (2) allow epithelial layer formation and tear film, (3) allow corneal innervation without side effects, (4) avoid immunologic reactions and infections, and (5) adopt corneal functions such as optical refraction. The next section of this review will discuss some of the most popular and widely used biomaterials that can be important for KC treatment.

### 5.1. Acellular Corneal Stroma

The most successful and widely accepted treatment for KC is corneal transplantation where the damaged tissue is replaced with a human donor cornea. While this is popular, there are a several limitations as discussed above. Recently, developments in bioengineered corneal substitutes have been designed, including acellular matrices [185,186,187,188,189,190,191]. Corneal ECM is mainly collagenous and the obvious choice would be an acellular collagen ECM which can be successfully implanted. This has been developed and tested mainly in animals [187,188,191,192] where the ACS relies on repopulation by host cells to restore corneal function and therefore vision. Griffith and co-authors reported ACS transplantation in humans [193] with the highest corrected visual acuity of 0.4 achieved. However, there were problems with these constructs, such as post-surgical astigmatism developed by 60% of the patients due to degradation of the biomaterial. Fish-scale collagen ECM is a different type of ACS and it has only been recently investigated in ocular research [194,195,196,197,198]. Briefly, it is a naturally occurring collagen Type I obtained from scales of the tilapia fish. Their scales consist of highly organized, parallel arranged collagen fibers that are packed in layers oriented approximately 90 degrees, mirroring a human cornea [194,195,196]. It has been shown that this ECM can support the growth of corneal cells *in vitro* with high levels of oxygen permeability [197]. In addition, the fish-scale ECM is shown to have minimum inflammatory responses *in vivo* [198]. Recently, van Essen and co-authors [194] reported promising results when the fish-scale ECM was used in a rat anterior lamellar keratoplasty. One of the latest technologies is the supramolecular 4-arm-PEG-(POG) biomaterial which shows great promise as a drug carrier in corneal diseases and defects [199]. While the ACS solution is by no means ready, it represents an interesting option for KC patients that require transplantation. With a shortage of corneal donor tissue it is vital that we consider alternative solutions. 

### 5.2. Collagen Equivalents

There is a huge variety of collagen models for corneal applications. Collagen has been used for years in many applications and holds great potential for many diseases. In KC, it is obvious to consider a partial transplantation with a collagen ECM. Here, we review some of the most promising systems available that might help in the KC treatment in the future. In its simplest form, a cornea equivalent consists of a collagen stromal ECM with corneal keratocytes seeded in it and covered with an epithelium layer [200,201,202,203]. Parnigotto *et al*. [201] mixed keratocytes with collagen extracted from rat tails and seeded a layer of epithelial cells on top, letting the co-culture grow for 7 days. The results showed a good epithelial layer with expression of known differentiation markers such as AE5. Germain [202,203] constructed a corneal equivalent with 4–5 layers of epithelium following culture for 3 days on top of collagen ECM with keratocytes. In that study, staining of anti-integrin β1 was shown in the basal cells, mimicking normal corneas *in vivo*. Other integrins reported included α3, α5, and α6. As an alternative to the rat tail collagen ECM, Orwin and Hubel [200] developed a co-culture model using bovine Type I dermal collagen. In that study, both epithelial-stroma and endothelial-stroma equivalents were reported.

As natural development, several attempts have been made to develop a more reliable corneal equivalent that mimics the entire human cornea *in vivo* and consist of all layers: corneal epithelium, stroma, and endothelium. So far, three-dimensional corneal equivalents with primary bovine corneal cells [204,205,206], primary rabbit corneal epithelial and endothelial cells, immortalized mouse corneal endothelial cells [207], primary corneal pig cell [208,209,210,211], primary human corneal epithelial cells and fibroblasts, immortalized human endothelial cells [212], and immortalized human corneal cell lines [213,214,215] have been reported. Whole corneal replacement is not necessary for KC treatment, and almost certainly an epithelium-stroma co-cultured ECM is the most likely target for KC transplantation. If developed, this could provide ways for treating KC defects and at least partially replace corneal transplantation. Clearly, there are questions about the viability of the constructs, such as survival of the cells once transplanted, cell type to be transplanted, cell source, and graft failure. These are only a few of the concerns that need to be addressed before a clear solution can be found.

### 5.3. Polymers

The ECM is a critical part of every tissue/organ and it provides physical support to tissues and defines cellular behaviors and tissue functions. The other popular approach in tissue engineering is the use of polymers. There are two main categories: biopolymers and synthetic polymers [216]. The main difference between the two is in their structure [217]. Biopolymers are polymers produced by living organisms and their structures are predetermined based on their physical characteristics [216,218,219]. On the other hand, the synthetic polymers are human-made and their properties are well controlled [217,220]. They have superior mechanical and physical properties and because they are human-made, are easy to produce in large batches and maintain reproducibility. The problem with them is that they tend to lack biocompatibility and therefore result in inflammatory responses, rejections by the host, and fibrosis. In cornea, the most well-known synthetic product is keratoprostheses (KPros) [221,222,223,224,225,226,227] which are designed to replace the central portion of an opaque cornea. KPros have been used for many years with relatively high success rates. A recent alternative has been the AlphaCor [228,229,230]. AlphCor is a transparent poly9hydroxyethyl methacrylate (pHEMA) sponge that allows cellular ingrowth. While this is a promising polymer, and it could potentially be used for KC treatment to replace cornea thickness and allow cell migration, there are significant problems, such as the non-existing re-epithelialization and nerve regeneration. Perhaps, the most promising report of polymers with adequate transparency for corneal repair and good cell ingrowth are from Bruinning *et al*. [231] who reported a free-radical polymerization of butyl methacrylate, hexaethyleneglycolmethacrylate, and a dimethacrylate cross-linker. 

In human cornea application, the most dominant biopolymers are collagen I and collagen V, which are the base of most bioscaffolds developed today. Griffith *et al*. [232,233] fabricated stromal ECM from glutaraldehyde cross linked collagen/chondroitin-6-sulfate hydrogels. The authors reported acceptable morphological characteristics; however, the physical properties of the material were inadequate for transplantation. As an improvement to their original model, the authors later reported a terpolymer named poly(*N*-isopropylacrylamide-co-acrylic acid-co-acryloxysuccinimide) or PNiPAAm-co-AAc-co-ASI [187,234]. These resulting hydrogels were adequate for transplantation and when tested in pigs, allowed epithelial overgrowth, stromal cell ingrowth, and functional nerve plexus [187].

## 6. Future

Biomaterials are expected by scientists to function as cell scaffolds to replace damaged or injured tissue/organ. The composition and properties of biomaterials used for tissue engineering varies significantly between individuals and tissues. Ultimately, the goal is to replace or regenerate the damaged tissue without any complications for the host. In the cornea, the major challenge is to replace and maintain the transparency properties of the original tissue. The complex structure of a dense and highly organized collagen ECM in the human cornea is a challenge for biomaterials. In KC, there are three main characteristics of the disease: (1) cornea thinning, (2) corneal bulging, and (3) corneal scarring. In order to apply tissue engineering techniques, there are several characteristics that the new biomaterial should provide. It should have similar mechanical and physical properties to the host tissue, replaces the stroma thickness while it allows for host keratocytes to migrate and repopulate it, and at the same time be friendly to the epithelial cells so they can grow on top of the graft. Of course, cell-seeded biomaterials are also possible and could be transplanted with keratocytes and epithelial cells. In this case, the main challenge would be to avoid graft rejection and any immunological responses by the host tissue. Conventional biomaterials are designed and fabricated relatively easily these days and have found applications in a variety of tissues. One of the most popular collagen-based ones is the animal-derived collagen and poly(glycolic acid) (PGA). Despite the progress, however, further refinement of current biotechnology and tissue engineering techniques is necessary. In order for our biomaterials to mimic the native tissue or organ at the nanoscale level and be able to provide biocompatibility, further more intelligent biomaterial development is necessary. In KC and in any ocular trauma defect, such a biomaterial would instantly find space in surgical rooms. Being able to restore vision is vital for quality of life. Corneal transplants are currently available and have great success; however, the cornea donor shortage is a major problem. This is especially true for third world countries or places where they do not have access to these tissues due to legislation, logistics, or religion. 

One area of research that remains to be explored further is the area of embryonic stem cells (ESC). Whether we are talking about injecting these cells or combining them with a substrate for transplantation, these cells have properties that can be proven vital in tissue engineering advancements. In KC, stem cells have not been employed or tested yet. Can these cells differentiate to keratocytes? Can they repair the lost corneal thickness? Can they restore its physical properties? Can they do it on their own or is a substrate is required? If yes, which substrate is the best for these cells to thrive? Autogenous ESCs would be ideal in order to minimize chances of host rejection and immune responses; however, this is not always available due to harvesting techniques and phenotype control. Use of xenogeneic or allogeneic cells requires even more attention since it is vital to shut down immunological avenues before transplantation. 

Still, great interest and potential remains for developing new, “smart” biomaterials that can be used with or without cells as implants to stimulate and enhance regeneration. Based on the composition of the cornea, the obvious choice of a biomaterial would be collagen based. However, any advancement would be well-received in combination or with modifications for promoting corneal regeneration. 

## 7. Conclusions

The systematic study of the physical and biochemical effects and capabilities of the available biomaterials as corneal replacement demonstrates their potential for future use in KC disease as well as in other corneal defects. The availability of an easy obtainable, cost-effective, and biocompatible biomaterial can have a high impact on treating KC and reduce the shortage of donor corneas. The arrival of CXL has definitely changed the KC treatment standards; however, there is still a large number of people that will require corneal transplantation and can benefit from the development of a biomaterial.
